# Neural Mechanisms of Choice Diversification

**DOI:** 10.3389/fnins.2020.00502

**Published:** 2020-06-03

**Authors:** Linda E. Couwenberg, Maarten A. S. Boksem, Alan G. Sanfey, Ale Smidts

**Affiliations:** ^1^Rotterdam School of Management, Erasmus University Rotterdam, Rotterdam, Netherlands; ^2^Donders Institute for Brain, Cognition and Behavior, Radboud University Nijmegen, Nijmegen, Netherlands; ^3^Behavioural Science Institute, Radboud University Nijmegen, Nijmegen, Netherlands

**Keywords:** decision-making, diversification, fMRI, novelty-seeking, satiation, ventral striatum, ventromedial prefrontal cortex

## Abstract

When asked to select several options at once, people tend to choose a greater diversity of items than when they are asked to make these selections one at a time. Using functional magnetic resonance imaging (fMRI), we provide novel insight into the neural mechanisms underlying diversification in portfolio choices. We found that, as participants made multiple selections from a menu of different options, the current state of their choice portfolio (i.e., the previously selected options) dynamically modulates activity in the neural valuation system in response to the options under evaluation. More specifically, we found that activity in the ventral striatum (VS) decreases when the option has already been selected (“satiation”), while activity in the ventromedial prefrontal cortex increases when other options have previously been selected (“novelty-seeking”). Our findings reveal two processes that drive diversification in portfolio choices, and suggest that the context of previous selections strongly impacts how the brain evaluates current choice options.

## Introduction

We frequently find ourselves in situations that require us to make multiple simultaneous selections from an often wide array of available options. For instance, we may decide to go to the supermarket on the weekend to buy several tubs of yogurt in anticipation of our weekly consumption. Research has shown that when asked to select several options at once for future use, people tend to choose a greater diversity of items than when they are asked to make these selections one at a time (i.e., choosing one tub of yogurt each day; e.g., Simonson, 1990; Read and Loewenstein, 1995). This tendency to diversify a choice portfolio typically leads to the selection of alternatives that are not usually purchased (Simonson and Winer, 1992), and the selection of relatively more “virtues” than “vices” (Read et al., 1999a). Interestingly, people are even willing to even forgo preferred options, making suboptimal choices, in order to construct choice portfolios with greater diversity (e.g., Read et al., 2001). For example, when selecting several tubs of yogurt, we may not only select our favorite flavor (i.e., strawberry), but also a less liked option (i.e., banana). This diversification phenomenon in choice behavior has been robustly demonstrated in various domains, such as food or movie selection, and similar patterns have been documented when allocating continuous resources (such as money) across a set of alternatives. For instance, people tend to diversify retirement savings relatively evenly across a set of possible investment instruments (Benartzi and Thaler, 2001), irrespective to some degree of return rates of each.

Despite the pervasiveness of daily life situations in which multiple selections are required, and the demonstrated profound consequence of diversification on choice outcomes, little is known about the mechanisms that drive this process. Insights into the neural mechanisms underlying these decisions are therefore important in advancing our understanding of this ubiquitous phenomenon.

According to the classic utility maximizing framework (e.g., Von Neumann and Morgenstern, 1947), a decision-maker first determines the utility of each available option, and then selects that option with the greatest utility. However, as people proceed through a series of choices, the state of their choice portfolio accordingly changes with each additional selection. To explain diversification, we propose that, in response to these changes, the utility of the available options in the choice set is updated dynamically. More specifically, we hypothesize that (1) the utility of an option decreases when it has previously been selected, this making it less likely to be added again, and/or (2) the utility of a non-chosen option increases when alternative options have already been added to the portfolio, which in turn leads to a greater chance of it being selected. Both of these proposed mechanisms could independently drive diversification. However, while the first hypothesis suggests a (“passive”) mechanism reflecting diminishing marginal utility (“satiation”; e.g., McAlister, 1982), the second hypothesis points to an intrinsic appreciation of change (“novelty-seeking”; e.g., Venkatesan, 1973).

Previous research on how the brain computes and represents choice utility has identified several neural areas that appear to carry a domain-general utility, or “value,” signal. These areas, often termed the “valuation system” (Bartra et al., 2013), include dopamine rich regions such as the ventral striatum (VS) and the (ventro)medial prefontal cortex [(V)MPFC; e.g., Knutson and Cooper, 2005; Delgado, 2007; Levy and Glimcher, 2012]. Although previous research using functional magnetic resonance imaging (fMRI) has been primarily concerned with exploring single choices made in isolation, a relevant insight is that value representation in the brain appears to be context-dependent (e.g., Plassmann et al., 2008; Seymour and McClure, 2008; De Martino et al., 2009), suggesting that a change in context due to previous selections may affect valuation of currently evaluated items.

Taken together, we hypothesize that as people make multiple selections from a given choice set, the state of one’s choice portfolio (i.e., the history of previously selected options) will dynamically modulate activity in the neural valuation system, leading—through either (or both) a “satiation” and “novelty-seeking” mechanism—to the commonly observed phenomenon of diversification of choice. We investigated this question, and the proposed mechanisms of interest, by scanning participants using fMRI while they made a series of product choices.

## Materials and Methods

### Participants

Forty-five participants completed the study. All provided written informed consent and were financially compensated via either a flat fee (30 euro) or study credits for completion of the task. In addition, all participants received one or more prizes (see below) in addition to this participation fee. Exclusion criteria included self-reported claustrophobia, neurological or cardiovascular diseases, psychiatric disorders, regular use of marijuana, use of psychotropic drugs, metal parts in the body, or any dietary restrictions (as many stimuli in the task were food items). Four participants were excluded due to excessive movement (>3 mm) during fMRI data acquisition. Data are therefore reported from 41 participants (13 men and 28 women, M = 22.73 years, SD = 3.28, range = 18–34 years, all right-handed). The study was approved by the local institution’s ethics committee.

### Stimuli

We selected 40 product categories, each incorporating five different products, to present as choice sets in the task. The majority of the product categories (i.e., 26 out of 40) consisted of food items (e.g., noodles, soup, or cereal). The remaining categories consisted of a variety of non-food items, such as socks, mugs, or hand soap. Within each category, the products were of the same brand and were priced similarly, but differed in terms of flavor, scent, or color (e.g., five different flavors of instant noodles). Participants’ liking scores for each of the 200 products was assessed on an 11-point slider scale with decimal accuracy (0 = “*I don’t like this product at all*,” 10 = “*I really like this product*”) in an online survey before the scanning session. In this survey, the products were presented per category, such that the five products per category were rated on the same page, ordered randomly. Based on these liking ratings, we ranked the products within each category for each participant individually. We ranked equally liked items (i.e., up to the second decimal) in random order. In order to select the most desirable set of stimuli for each participant, we excluded five product categories in which the most liked product had a liking rating lower than 4 on the 11-point scale. In case we were not able to exclude five product categories using this rule, we excluded categories with the greatest similarity in liking ratings. We used these excluded product categories in the filler trials. The remaining 35 product categories were presented in the trials of interest.

### Task

We developed a novel paradigm to study the neural mechanisms underlying diversification in choice behavior, optimized to disentangle the hypothesized “satiation” and “novelty-seeking” mechanisms. Participants were informed that they would participate in a study examining reaction time accuracy. Each series of choices in our experiment was preceded by a simple time-estimation task (Boksem et al., 2011), in which participants saw a grayscale visual cue that changed to color after 1000 ms. Participants were instructed to press a response button exactly 1000 ms after this color change. Responses were considered correct when reaction times fell within an allowable time-interval. Participants continued onto a new time-estimation trial if their response did not fall within this time-interval (i.e., either too fast or too slow). After a correct response, participants began the choice part of the task (i.e., the task of interest) in order to select their prize(s). The purpose of this time-estimation task was to both maintain engagement throughout the task, and, importantly, to create a context for making a series of product choices. Participants were instructed that one of the time-estimation trials they played would be randomly drawn at the end of the experiment and that—if they had been successful on that trial—they would receive the prize(s) they had selected after that specific trial.

Upon entering the choice part of the task, participants viewed a screen consisting of a choice set of five products from a specific product category (e.g., five different flavors of instant noodles). One of these five products was highlighted, and participants were instructed to passively evaluate this product for 3000 ms (i.e., they were asked to consider “*Do you want this product?*”). This evaluation screen constituted the time window of interest for the statistical analyses, and its onset was jittered (3000–5000 ms). Participants were then asked to make their decision to either accept or reject this specific product using a button box (placed in their dominant hand). The task advanced right after the participant made their choice, with a maximum response time of 2500 ms. To stimulate participants to only accept products they really wanted on each specific choice occasion, participants did not know in advance how many total products per choice set they would get to select. That is, every decision to accept could be their final opportunity to select a product from the current category. Participants could reject products an unlimited number of times (e.g., they could choose to wait, at some risk, for their highest preference product to be offered). In each of the 35 choice trials of interest for our analyses, participants could select a total of three prizes. A different choice set (i.e., product category) was used in each of these trials. To ensure that each accept-decision in these trials was consequential, participants could select a total of one, two or four prizes in the remaining 14 filler trials. In these filler trials, each of the five excluded product categories was repeated two or three times. The filler trials were distributed pseudo-randomly throughout the whole experiment, such that the trials of interest were alternated with filler trials.

Once a product was accepted it appeared in a “basket,” which was always visible below the choice options. The main goal of the study was to investigate the influence of the dynamic state of this “basket” (i.e., the products it contained during the evaluation phase) on neural responses and subsequent choice. After accepting a product, participants either evaluated another product, or continued with the next time-estimation trial (i.e., when the total number of selections for the current category was reached). If they rejected a product, participants continued to evaluate products, until they accepted one.

**FIGURE 1:**
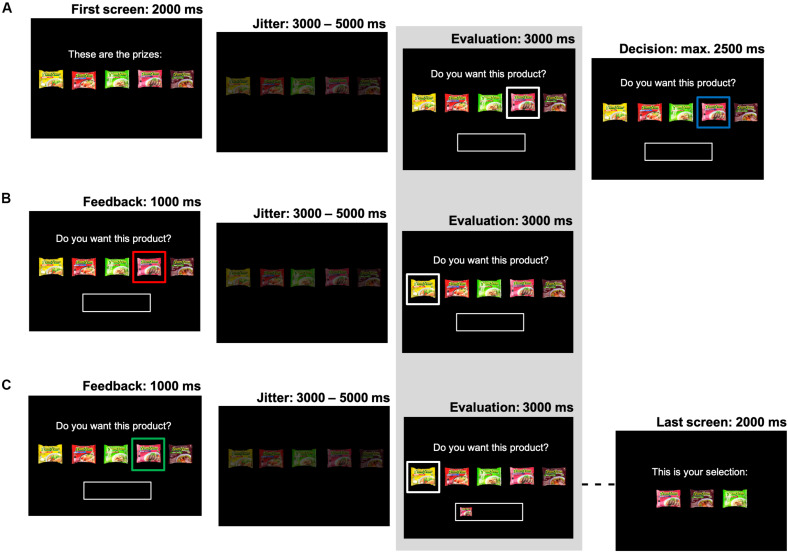
Task design. The structure of the choice task is presented. Each picture represents a screen in the experiment. The evaluation screen (indicated by the shaded area) constituted the screen of interest for the analyses and its onset was jittered (3000–5000 ms). **(A)** Each choice set consisted of five products. One product was offered at the time (highlighted with a white box). This focal product was evaluated (cued by a blue box), and then accepted or rejected. **(B)** When the product was rejected (red box), another product was evaluated. **(C)** When a product was accepted (green box), it then appeared in the basket. Products were evaluated until three products were selected. After the last screen, a new time-estimation trial started.

The order in which the products were to be evaluated was first based on the product rankings, and then dynamically updated based on the participants’ decisions for that specific category. This allowed us to control the number of observations of interest to distinguish between the “satiation” and “novelty-seeking” mechanisms, without restricting participants’ freedom of choice. That is, within each series of choices, we presented participants with a previously accepted product for a second time in order to test whether choice and neural valuation for this option would decrease (i.e., “satiation”). Additionally, we exposed participants to a previously rejected product for a second time, in order to test whether choice and neural valuation for a previously non-selected option would increase once different products had been selected in the meantime (i.e., “novelty-seeking”). We optimized the sequence of product evaluations to maximize the number of these type of observations, by presenting lower ranked products first (to elicit a “reject” decision), and then higher ranked products (to elicit an “accept” decision). A previously accepted product was then presented again (now with this same product in the “basket”), and a previously rejected product was only presented again once there was another accepted product in the “basket.” As this product presentation sequence was dependent on the participants’ decisions in the task, the number of repeated exposures to accepted or reject products could differ by product category and participant (see section “fMRI Data Analysis” for details). Participants were free to either make the same choice [accept (reject) a previously accepted (rejected) product again] or change their mind [accept (reject) a previously rejected (accepted) product]. In the filler trials, the sequence in which the products were presented followed the rank order, starting with the highest ranked product. See Figure 1 for a pictorial overview of the choice task.

### Procedure

At least 3 days before the fMRI scanning session, participants completed an online survey in which we assessed their liking for each of the products presented in our task. Upon arrival in the fMRI lab, participants performed two practice sessions. In the first session, participants practiced the time-estimation task. In this practice, which consisted of 20 trials, we used a minimum and a maximum response time to determine an initial allowable response time-window (i.e., 700–1300 ms). If participants responded within this time-window, this interval was shortened by 50 ms; if they responded either too quickly or too slowly, the interval was lengthened by 50 ms. The resulting interval after the last practice trial was used as the time-window for the time-estimation task in the experiment, thus individually calibrated for each participant. This time-window was covertly adjusted throughout the experiment in order to ensure a sufficient number of hits (and thus choice trials). If participants responded within the allowable time-window, the interval was shortened by 10 ms; if they responded either too quickly or too slowly, the interval was lengthened by 90 ms. So, although the proportion of hits (±90%) and misses (±10%) was controlled, the feedback was contingent upon participants’ actual performance.

In the second practice session, participants became familiar with the choice task. After these practice sessions, participants entered the scanner and practiced with the button box. The experiment, which was programmed and presented in Presentation software (Version 16.3^1^), was one continuous run of approximately 45 min while fMRI data were being collected. After the experimental task, we collected the anatomical scan. Finally, participants were thanked and paid. For the bonus payment, we only selected from hit trials, although participants were made to believe that both hit and miss trials in the time-estimation task could be randomly drawn, as the number of hits and misses was controlled. Each participant was therefore awarded up to four of their selected product(s) in addition to the participation fee.

### fMRI Data Acquisition

Imaging was performed using a 3-T head-dedicated MRI system (Siemens Magnetom Skyra). Functional MRI images were acquired using a 32-channel head coil, with a standard multi-echo imaging pulse T2^∗^-weighted sequence [field of view (FOV): 224 mm; 64 × 64 matrix; repetition time (TR): 2250 ms; echo times (TEs): 9.4, 21.2, 33, 45, 56 ms; flip angle: 90°, 0.5 mm slice gap]. Using a multi-echo sequence provides a better signal-to-noise ratio for brain areas susceptible to drop-out, while allowing for scanning of the whole brain (Poser et al., 2006). Thirty-five ascending slices were acquired (thickness: 3.0 mm; voxel size: 3.5 × 3.5 × 3.0 mm) from the whole brain. High-resolution anatomical T1-weighted image (MPRAGE; 192 slices; TR: 2300 ms; voxel size: 1 × 1 × 1 mm) was acquired for anatomical localization. Participants’ heads were lightly restrained with tape loosely placed between their head and the coil within the scanner in order to limit movement during image acquisition.

### fMRI Data Analysis

Analyses on the brain data were performed using SPM12 (Statistical Parametric Mapping; Wellcome Department, London, United Kingdom). Prior to preprocessing, we combined and realigned the five read-outs acquired via the multi-echo sequence by using standard procedures described by Poser et al. (2006). Preprocessing consisted of realignment, slice-time correction to the middle slice, segmentation of the functional and anatomical image, co-registration of the functional images to the anatomical images, and normalization to the Montreal Neurological Institute (MNI) template using the segmentation parameters. Functional images were then smoothed with a Gaussian kernel of 8 mm full-width at half-maximum (FWHM). The first 30 volumes, acquired prior to task initiation, were used to estimate the weighted ET per voxel for optimal echo combination (Poser et al., 2006) including allowing T1 equilibration effects, and discarded from the analysis. Motion parameters were stored and used as nuisance variables in all generalized linear model (GLM) analyses. The task consisted of a single run of approximately 45 min; a standard high-pass filter (cut-off 128 s) was used in the analyses to account for possible slow-frequency drifts.

For the statistical analyses of the brain data, we first ran first-level GLMs to identify the brain regions related to the choice to accept a product (the “valuation network”). The model consisted of two regressors of interest (1. “accept,” 2. “reject”) that were time-locked to the evaluation screens of the choice part of the task, with “accept” and “reject” referring to the subsequent choice outcome. We performed a *t*-test at the group-level, contrasting the two regressors to find the unique activations related to the decision to “accept” (vs. “reject”). The reported main results exceed the statistical threshold of *p* < 0.05 FWE corrected on the cluster-level.

Next, we assessed how the dynamic state of the choice portfolio modulated activity in the brain regions associated with the decision to accept a product. To this end, we constructed regions-of-interest (ROIs) within the most significant brain regions (3 mm radius spheres around the most significant peak voxels) from the “accept” vs. “reject” contrast. We extracted parameter estimates from the selected ROIs with MarsBaR (Brett et al., 2002), using first-level GLMs with a separate regressor for each observation, time-locked to the evaluation screen. To test our “satiation” hypothesis, we only selected choice options that were accepted the first time they were evaluated (“satiation T1”), and also evaluated a second time (“satiation T2”). This subset of observations included a total of 1455 pairwise comparisons across all participants, with at least one pairwise comparison in each of the 35 choice portfolios per participant (median number of pairwise comparisons per participant = 35; minimum = 35; maximum = 39). To test our “novelty-seeking” hypothesis, we selected a different subset of choice options that were rejected the first time they were evaluated (when the “basket” was empty; “novelty-seeking T1”), and then evaluated a second time once other choice options were selected in the meantime (“novelty-seeking T2”). This subset of observations included a total of 856 pairwise comparisons across all participants, with at least one pairwise comparison in on average 47.6% of the 35 choice portfolios per participant (median number of pairwise comparisons per participant = 21; minimum = 7; maximum = 36). For both subsets, we tested for pairwise differences in signal change using repeated measures ANOVAs in R software^2^, with the effect of time (T1, T2) nested within participants.

## Results

### Behavioral Data

The data show that participants indeed diversified their choice portfolio in the majority of the product categories. Overall, of the total of 1435 choice portfolios of three products each, 47.2% consisted of two unique items, and 33.2% of three unique items. A minority of the choice portfolios (19.6%) consisted of three of the same items, and thus were not diversified. In addition, each individual participant diversified a substantial number of their 35 choice portfolios (median = 30; min = 14; max = 35).

To test if participants diversified because of “satiation” or “novelty-seeking,” we ran non-parametric Wilcoxon signed-rank tests to compare probabilities of selecting an item, dependent on particular states of the basket. To make sure that we compared products of similar *a priori* liking, we created bins of items of homogeneous relative preference based on rank score. We selected Rank 1 and Rank 2 items for our “satiation” test because these occurred most often in the task to maximize the number of “satiation” observations. We selected Rank 3 and Rank 4 for novelty-seeking because these occurred most often in the task to maximize the number of “novelty” observations. We omitted the least liked items (Rank 5) items because the limited number of observations. To test our “satiation” hypothesis, we ran a Wilcoxon signed-rank test, comparing the probability of accepting a product given that it is not in the basket with the probability of accepting a product given that it is in the basket, indicating a significant decrease in probability (*P*_T__1__–Accept_ = 0.863, *P*_T__2__–Accept_ = 0.552, *Z* = −5.073, *p* = 0.000). To test our “novelty-seeking” hypothesis, we ran another Wilcoxon signed-rank test, comparing the probability of accepting a product given that the basket is empty with the probability of accepting a product given that other items (but not the current item) are in the basket, showing a significant increase in probability (*P*_T__1__–Accept_ = 0.331, *P*_T__2__–Accept_ = 0.453, *Z* = −4.062, *p* = 0.000). These results indicate that as people make multiple selections from a given choice set, the state of one’s choice portfolio (i.e., the history of previously selected options) leads—through both “satiation” and “novelty-seeking”—to diversification of choice.

In addition, of the subset of 1455 pairwise observations selected to test the “satiation” hypothesis on the neural data (i.e., choice options that were accepted the first time they were evaluated, and also evaluated a second time), the item was rejected at T2 in 40.7% of the cases (reject rate per participant: median = 34%, min = 0%; max = 91%). In 67.2% of those cases, the rejected item was the most preferred item (i.e., the item with the highest *a priori* liking score). Of the subset of 856 pairwise observations selected to test our “novelty-seeking” hypothesis on the neural data [i.e., choice options that were rejected the first time they were evaluated (when the “basket” was empty), and then evaluated a second time once other choice options were selected in the meantime], the item was accepted at T2 in 19.6% of the cases (accept rate per participant: median = 14.2%, min = 0%; max = 47%). In 52.4% of those cases, this was the third item added to the “basket,” and the majority of those selections included the third ranked item (75.6%).

We further hypothesized that if people diversified, they would be willing to accept products with lower liking ratings than their most preferred product. We ran a linear mixed model (with random intercepts for individuals) to test if liking ratings of the most liked product and the selected products in a given product category are significantly different. The results show that this difference was highly significant [M_Δ__Highest Liking –__Mean Liking (Accepted)_ = 0.737; *t*(40) = 13.82, *p* = 0.000]. Moreover, we found that the higher the variety (number of unique items) across choice portfolios, the higher this difference in liking (Pearson’s *r* = 0.565, *p* = 0.000).

In summary, these findings suggest that utility of options in the choice set is indeed modulated by the dynamic state of the choice portfolio, and they provide clear behavioral indications of both “satiation” and “novelty-seeking” processes.

### fMRI Data

#### Neural Correlates of Choice

**FIGURE 2:**
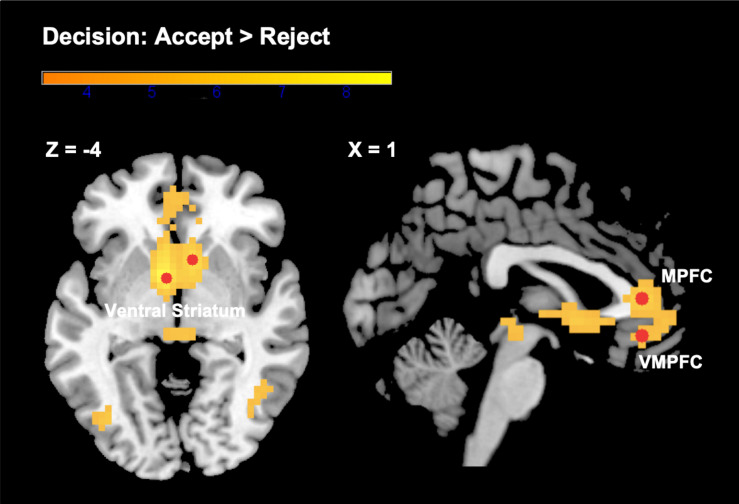
Brain activations in the VS, MPFC, and VMPFC from the contrast “subsequent decision: accept > reject.” Color bar represents *t*-statistics. Selected ROIs (3 mm radius spheres around the most significant peak voxels) are depicted in red. See Table 1 for more details not shown here.

We found expected brain activation patterns in response to evaluated choice options that were subsequently accepted, as compared to those that were evaluated and rejected. Areas of increased activations for accepted as opposed to rejected options included a cluster spanning the VS (bilateral nucleus accumbens), the MPFC, and the VMPFC (see Figure 2). Other regions identified by this contrast were the middle temporal gyrus, inferior frontal gyrus, middle occipital gyrus, midbrain, and precuneus. Although we were primarily interested in the neural activity related to the decision to accept (“positive valuation”), we also analyzed the opposite contrast (“reject” > “accept”). When participants subsequently rejected the choice option under evaluation, we found increased activity in the supramarginal gyrus extending into the putamen, superior temporal gyrus, middle and inferior frontal gyrus, angular gyrus, calcarine, and cerebellum. See Table 1 for a detailed overview of our findings.

**TABLE 1:** Brain activations for subsequent decision (accept, reject).

Anatomy	Hemisphere	MNI			Cluster size	*Z*
			
	L/R	*x*	*y*	*z*	[voxels]	
**(A) Accept > reject**						
VS/MPFC/VMPFC					435	
VS (NAcc)	L	−6	4	−4		6.47
VS (NAcc)	R	8	14	−4		5.25
MPFC	R	1	35	7		4.65
VMPFC	R	1	35	−10		3.72
Middle temporal gyrus	R	43	−70	7	137	4.81
Middle occipital gyrus	L	−38	−74	4	75	4.74
**(B) Reject > accept**						
Supramarginal gyrus/putamen	L	−52	−24	21	1386	6.33
Superior temporal gyrus	R	68	−21	4	212	4.88
Middle frontal gyrus	R	36	42	32	457	4.74
Angular gyrus	R	57	−60	28	95	4.66
Middle frontal gyrus	L	−34	38	28	89	3.85

*Note: (A) Brain activations for subsequent decision (accept > reject). (B) Brain activations for subsequent decision (reject > accept). All reported activations exceeded the threshold of *p* < 0.05 FWE corrected on the cluster-level. *Z*-values for each peak are given. Abbreviations: L = left; R = right; VS = ventral striatum; NAcc = nucleus accumbens; MPFC = medial prefrontal cortex; VMPFC = ventromedial prefrontal cortex. *N* = 41.*

#### Choice Portfolio Effects

In order to test how valuation of options in the choice set is modulated by the current state of the choice portfolio, and to further tease apart the potential proposed “satiation” and “novelty-seeking” mechanisms, we focused subsequent analyses on the cluster of neural activity most significantly correlated with the decision to accept. This cluster spanned regions typically related to positive valuation in previous studies (the VS, the MPFC, and the VMPFC). We constructed three ROIs around the most significant peak voxels within this cluster: in the VS [bilateral; *x*: −6, *y*: 4, *z*: −4 and *x*: 8, *y*: 14, *z*: −4 (left and right averaged)], the MPFC (*x*: 1, *y*: 35, *z*: 7), and the VMPFC (*x*: 1, *y*: 35, *z*: −10), and extracted parameter estimates for the evaluation phase of each trial.

**FIGURE 3:**
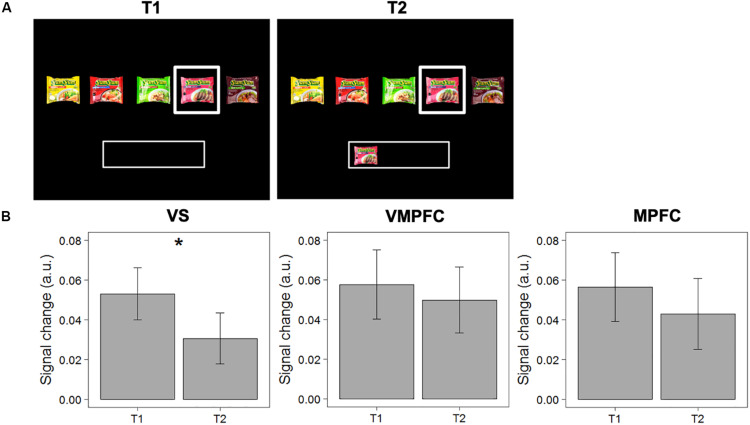
Test of the “satiation” hypothesis. **(A)** Example of a pairwise comparison of the first time an option was evaluated (T1) and the second time the same option was evaluated when this option was already in the “basket” (T2). **(B)** Differences in signal change between T1 and T2 in the VS, VMPFC, and MPFC. Error bars represent standard errors of the mean. * The difference in signal change between T1 and T2 is only significant in the VS [*F*(1,40) = 5.188, *p* = 0.028].

First, we tested the “satiation” hypothesis, which posits that activity in the valuation network decreases when the evaluated option has previously been selected. We compared signal change in response to choice options evaluated for the first time (and subsequently accepted; T1), with the same option evaluated a subsequent time when this item was already in the “basket” (T2). Repeated measures ANOVAs reveal that activity in the VS decreases significantly between T1 and T2 [MΔT2-T1 = −0.022; *F*(1,40) = 5.188, *p* = 0.028]. This difference between T1 and T2 showed similar patterns in the MPFC and VMPFC, though did not reach significance in these other areas [*F*(1,40) = 0.618, *p* = 0.436; *F*(1,40) = 0.193, *p* = 0.663, respectively], see Figure 3 for details. These results show that valuation, particularly in the VS, for a particular choice option decreases when it has previously been selected.

It should be noted that, as we defined our ROIs based on the “accept” > “reject” contrast, comparing “accept” trials at T1 with “accept” *and* “reject” trials at T2 within these ROIs could potentially inflate the results. To check this, we ran a linear mixed model (with random intercepts for individuals) to test if the observed decrease in signal change in the VS is significantly different for items that were accepted or rejected at T2. The results show that this was not the case [*t*(1453) = −0.861, *p* = 0.38]. In addition, we analyzed whether the differences could be observed in a specific subset of pairwise comparisons of the same choice option with the same choice outcome (i.e., T1: “accept”; T2: “accept”). The results indeed show a decrease in activation in the VS, also for items that were accepted again (M_Δ__T__2__–T__1_ = −0.014), even though this difference did not reach statistical significance [*F*(1,40) = 1.216; *p* = 0.277]. It should be noted that this is a highly conservative test, as we do not necessarily hypothesize a difference within this particular subset of observations (we actually hypothesize “reject” decisions at T2).

**FIGURE 4:**
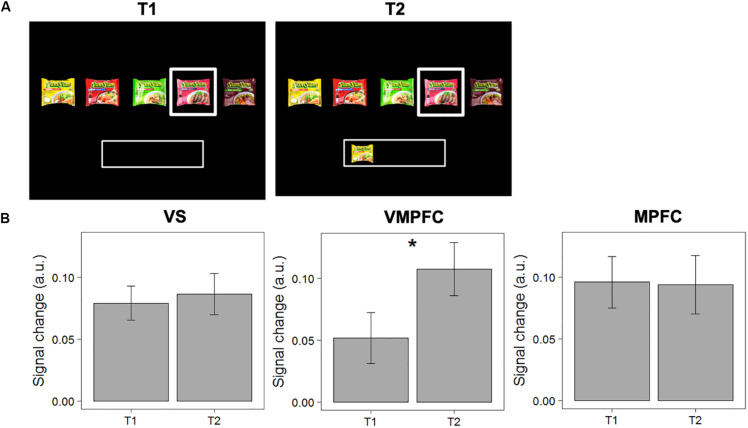
Test of the “novelty-seeking” hypothesis. **(A)** Example of a pairwise comparison of the first time an option was evaluated (T1) and the second time the same option was evaluated when another option was in the “basket” (T2). **(B)** Differences in signal change between T1 and T2 in the VS, VMPFC, and MPFC. Error bars represent standard errors of the mean. * The difference in signal change between T1 and T2 is only significant in the VMPFC [*F*(1,40) = 5.281, *p* = 0.027].

Second, we tested the “novelty-seeking” hypothesis, which suggests that activity in the valuation network for a non-selected option increases when alternative options have been already chosen. We compared signal change in response to choice options when evaluated for the first time with an empty “basket” (and subsequently rejected; T1), with the same choice option when evaluated a second time when other choice options were now in the “basket” (T2). We find that activity in the VMPFC increases significantly between T1 and T2 [MΔT2-T1 = 0.055; *F*(1,40) = 5.281, *p* = 0.027]. The difference between T1 and T2 was not significant in the VS [*F*(1,40) = 0.157, *p* = 0.694], nor in the MPFC [*F*(1,40) = 0.007, *p* = 0.933). See Figure 4 for details.

To account for the possibility of inflated results, we ran another linear mixed model (with random intercepts for individuals) to test if the observed decrease in signal change in the VMPFC is significantly different for items that were accepted or rejected at T2. The results show that this increase in signal change in the VMPFC is not significantly different for items that were accepted versus rejected at T2 [*t*(816.84) = 0.138, *p* = 0.891]. Moreover, we analyzed whether the differences could be observed in a subset of pairwise comparisons of the same choice option with the same choice outcome (i.e., T1: “reject”; T2: “reject”). The data show a significant increase in signal change in the VMPFC [M_Δ__T__2__–T__1_ = 0.057; *F*(1,40) = 4.088; *p* = 0.049]. Together, these analyses demonstrate that valuation for a current option increases when alternative items were previously selected, independent of the choice outcome at T2.

To assess to what extent the changes in neural response to “satiation” and “novelty-seeking” trials can be associated with distinct regions in the valuation network (VS or VMPFC), we ran a linear mixed model (with random intercepts for individuals) separately for “satiation” and “novelty-seeking” trials to test if the differences in signal change between the VS and VMPFC are significantly different. The results show that for “satiation” trials this difference did not reach significance [M_Δ__VS (T__2__–T__1__) – VMPFC(T__2__–T__1__)_ = −0.015, *t*(39.99) = −0.873, *p* = 0.388], while for “novelty-seeking” trials the neural response is indeed significantly stronger in the VMPFC as compared to the VS [M_Δ__VS (T__2__–T__1__) – VMPFC(T__2__–T__1__)_ = −0.048, *t*(855) = −2.421, *p* = 0.016].

## Discussion

In this study, we provide novel insights into the mechanisms underlying choice diversification in portfolios. We propose that, as people make multiple selections from a menu of different options, the current state of their choice portfolio (i.e., the history of previously selected options) dynamically influences the utility of the options in the choice set, represented in the brain’s valuation network. More specifically, we hypothesized that two different psychological mechanisms could drive diversification independently. People may diversify because (1) the utility of an option decreases when that option has been already selected (“satiation”) and/or (2) the utility of a non-selected option increases when alternative options have already been picked (“novelty-seeking”). We investigated how the neural valuation network might update the utility signal to enable these choice patterns.

Our behavioral data confirm that participants indeed diversify the majority of their choice portfolios. The choice data also demonstrate that some items were more likely to be rejected when they were already selected before, potentially indicating “satiation,” and that some items were more likely to be accepted once alternative items were selected, suggestive of “novelty-seeking.”

The neural data provide evidence that these portfolio effects on choice are driven by valuation processes. That is, we find that activity in both the VS (NAcc) and the VMPFC—brain regions also shown in previous literature to contribute to value-based decision-making (e.g., Knutson and Cooper, 2005; Delgado, 2007; Levy and Glimcher, 2012)—was modulated by the context of previously selected options. More specifically, our findings show that, most prominently, activity in the VS decreased in response to options if they had been previously selected, aligning with the “satiation” hypothesis. At the same time, we find an increase in activity in the VMPFC, in response to previously rejected options when other options have then been selected. This finding suggests that people also intrinsically have greater value for different or novel options as they are completing their choice portfolio, in line with the “novelty-seeking” hypothesis. Thus, our results suggest that both the “satiation” and “novelty-seeking” mechanisms can drive diversification, and are represented at the neural level by regions within the brain’s valuation network.

As we noted, in our analyses, we included all instances of a second viewing to test our “satiation” and “novelty-seeking” hypotheses. That is, to test the “satiation” hypothesis, we compared responses to items that were accepted the first time, and accepted or rejected the second time. Similarly, to test the “novelty-seeking” hypothesis, we compared responses to items that were rejected the first time, and accepted or rejected the second time. However, as we defined our ROIs based on the “accept” > “reject” contrast, comparing “accept” *or* “reject” trials at T1 with “accept” *and* “reject” trials at T2 within these ROIs could potentially inflate the results. We show that for “novelty-seeking” trials, the results hold when we only select a subset of items that were rejected at T1 and also at T2 (so avoiding comparing “accept” vs “reject” trials). For “satiation” trials, we show that, even though the decrease in signal change in the VS when comparing items that were accepted at T1 and subsequently accepted again at T2 was not smaller than for items that we rejected at T2, the direct contrast between T1 and T2 for items that were accepted in both cases did not reach significance. It should be noted that this is a highly conservative test, as we do not necessarily hypothesize a difference within this particular subset of observations (i.e., a decrease in valuation should often lead to “reject” decisions at T2). In addition, the observed effect for “satiation” is in the hypothesized direction, and the lack of significance could be due to the limited number of observations that remain for this contrast.

In previous neuroimaging research, increased striatal activity—specifically in the nucleus accumbens—has been related to the anticipation of reward (e.g., Knutson et al., 2000). Hence, decreasing neural responses in the VS to a previously selected choice option may indicate an anticipation that repeated exposure to the given option will be less satisfying. While the strict definition of “satiation” implies the diminishing marginal utility of an option after its repeated use or consumption (e.g., McAlister, 1982)—which therefore cannot predict diversification when making several choices at once—our findings do suggest that *anticipated* satiation, potentially encoded by the VS at the time of decision-making, could underlie choice diversification in portfolios. In a similar vein, research providing neurobiological support for marginal utility theory in a financial context has demonstrated that striatal activity in response to financial gains decreases in line with the increasing assets of individuals (Tobler et al., 2007). Note, however, that, although the decrease in valuation found here in response to previously selected options was most pronounced in the VS, it could not be reliably distinguished from the somewhat smaller decrease observed in the VMPFC, suggesting that (anticipated) satiation may be encoded rather broadly within the valuation system.

Our results suggest that the VMPFC might also play a different role in the context of portfolio choices, one more related to encoding the value of non-sampled options. Increased activity in the VMPFC has been related to value computation and executive control in previous literature. Consistently, the VMPFC is involved in predicting action outcomes, suggesting that this area encodes action–outcome associations in order to make selections according to the reward value ascribed to the respective actions (Hampton et al., 2006). For instance, the VMPFC has been found to encode a signal reflecting the comparison between current and alternative actions, incorporating both the subjective value of the current action as well as the opportunity cost of not selecting the alternative actions (Boorman et al., 2009, 2013). Thus, the VMPFC might be implicated in the assessment of whether or not it is worth adapting or maintaining decisions. While the VMPFC has been found to encode relative value of chosen options in related multi-alternative sequential choice tasks, such as foraging paradigms (e.g., Kolling et al., 2012), it should be noted that the present choice paradigm critically differs from these tasks in that there are no direct costs associated with choosing a different option, or feedback provided to indicate that there is a change in the actual value of the repeatedly selected item. The VMPFC has also been related to affective foresight, mediating mental simulations of the affective value of future outcomes (Bechara et al., 2000; Bechara and Damasio, 2005). In the current study, the observed VMPFC signal in response to an option that is different from previously selected options in a particular choice portfolio might reflect a motivation to change course, based on the predicted value of the outcome of that decision (e.g., more variety).

Taken together, our results show, to some extent dissociable, roles for the VS and the VMPFC in value-based portfolio choices. We propose that the VS might be more strongly involved in “simple” option-by-option valuation, with a decrease in responsivity reflecting anticipated satiation for the given option, while the VMPFC is (also) recruited for top-down control, with an increase in activity representing the high value of novelty or change. Triggered by different states of the choice portfolio, our findings suggest that these mechanisms can drive diversification via different processes.

Our results thus suggest that the bundle of the previous selections, the essential element that distinguishes portfolio choices from single choices made in isolation, can strongly impact how the brain values choice options. This indicates that making several selections together can prompt decision-makers to choose options that optimize the overall experience of the portfolio, instead of considering the experience of the options when taken in isolation (see also Read et al., 1999b). As reflected in our liking data, this can sometimes lead to seemingly “sub-optimal” choices, such as when a bundle consisting of a preferred and a somewhat less preferred option (e.g., strawberry and banana yogurt) is chosen over a bundle that consists of the preferred option twice (e.g., two tubs of strawberry yogurt). Our data here suggest that the interdependence of the (anticipated) experience of selected options might receive greater attention when making portfolio choices. The current research thus provides both behavioral and neuroscientific evidence of this interdependence, describing diversification behavior driven by both “satiation” and “novelty-seeking” mechanisms.

## Data Availability Statement

The original contributions presented in this study are publicly available available. This data can be found here: 10.25397/eur.12229211.

## Ethics Statement

The studies involving human participants were reviewed and approved by Donders Institute for Brain, Cognition and Behavior, Radboud University Nijmegen, Nijmegen, Netherlands. The participants provided their written informed consent to participate in this study.

## Author Contributions

LC, MB, ASa, and ASm designed the study. LC collected the fMRI data, analyzed the data, and wrote the manuscript. MB, ASa, and ASm provided input on the data analysis, interpretation of the results, and the final manuscript.

## Conflict of Interest

The authors declare that the research was conducted in the absence of any commercial or financial relationships that could be construed as a potential conflict of interest.

## Footnotes

1 www.neurobs.com

2 www.R-project.org
